# Current State of Digital Biomarker Technologies for Real-Life, Home-Based Monitoring of Cognitive Function for Mild Cognitive Impairment to Mild Alzheimer Disease and Implications for Clinical Care: Systematic Review

**DOI:** 10.2196/12785

**Published:** 2019-08-30

**Authors:** Antoine Piau, Katherine Wild, Nora Mattek, Jeffrey Kaye

**Affiliations:** 1 Gerontopole University Hospital of Toulouse Université Paul Sabatier Toulouse France; 2 Oregon Center for Aging and Technology Oregon Health and Science University Portland, OR United States

**Keywords:** technology, Alzheimer disease, cognition disorders, dementia, older adults, digital biomarkers, digital phenotyping, digital health

## Abstract

**Background:**

Among areas that have challenged the progress of dementia care has been the assessment of change in symptoms over time. Digital biomarkers are defined as objective, quantifiable, physiological, and behavioral data that are collected and measured by means of digital devices, such as embedded environmental sensors or wearables. Digital biomarkers provide an alternative assessment approach, as they allow objective, ecologically valid, and long-term follow-up with continuous assessment. Despite the promise of a multitude of sensors and devices that can be applied, there are no agreed-upon standards for digital biomarkers, nor are there comprehensive evidence-based results for which digital biomarkers may be demonstrated to be most effective.

**Objective:**

In this review, we seek to answer the following questions: (1) What is the evidence for real-life, home-based use of technologies for early detection and follow-up of mild cognitive impairment (MCI) or dementia? And (2) What transformation might clinicians expect in their everyday practices?

**Methods:**

A systematic search was conducted in PubMed, Cochrane, and Scopus databases for papers published from inception to July 2018. We searched for studies examining the implementation of digital biomarker technologies for mild cognitive impairment or mild Alzheimer disease follow-up and detection in nonclinic, home-based settings. All studies that included the following were examined: community-dwelling older adults (aged 65 years or older); cognitively healthy participants or those presenting with cognitive decline, from subjective cognitive complaints to early Alzheimer disease; a focus on home-based evaluation for noninterventional follow-up; and remote diagnosis of cognitive deterioration.

**Results:**

An initial sample of 4811 English-language papers were retrieved. After screening and review, 26 studies were eligible for inclusion in the review. These studies ranged from 12 to 279 participants and lasted between 3 days to 3.6 years. Most common reasons for exclusion were as follows: inappropriate setting (eg, hospital setting), intervention (eg, drugs and rehabilitation), or population (eg, psychiatry and Parkinson disease). We summarized these studies into four groups, accounting for overlap and based on the proposed technological solutions, to extract relevant data: (1) data from dedicated embedded or passive sensors, (2) data from dedicated wearable sensors, (3) data from dedicated or purposive technological solutions (eg, games or surveys), and (4) data derived from use of nondedicated technological solutions (eg, computer mouse movements).

**Conclusions:**

Few publications dealt with home-based, real-life evaluations. Most technologies were far removed from everyday life experiences and were not mature enough for use under nonoptimal or uncontrolled conditions. Evidence available from embedded passive sensors represents the most relatively mature research area, suggesting that some of these solutions could be proposed to larger populations in the coming decade. The clinical and research communities would benefit from increasing attention to these technologies going forward.

## Introduction

### Dementia and New Technologies

Interest in technologies as solutions for the challenges of dementia is high. Despite a plethora of technologies ranging from companion robots to fully functional smart home assessment environments, development and adoption has been slow or inconsistent [1]. In general, there is a wide spectrum of opinion about the utility of these technologies; these range from convinced *technophiles,* who believe that new technologies, particularly information and communication technologies (ICT) and artificial intelligence (AI), will revolutionize medicine, to skeptics or those not interested at all or who are even fearful of potential outcomes.

Among the most important areas that have challenged the progress of dementia care and treatment has been the assessment of those affected, those who are either at risk or presymptomatic, as well as those with clear, manifest symptoms [2,3]. At the root of this challenge is the need to identify symptoms and, most importantly, identify change in symptoms over time [3]. The latter is the essence of the diagnosis of dementia (ie, that there is a change from a prior state of normal cognition to a point where function is disturbed) [4,5]. This fact drives the basic approach that every clinician involved in mild cognitive impairment (MCI) and dementia assessment and care follows in their practice. It results in the need to assess, through careful history taking and neuropsychological assessment, whether a patient is experiencing change that reflects underlying neuropathology. It is vital to directing appropriate therapies [4,5].

### Digital Biomarkers Development

To aid in the more precise assessment of patients, clinicians increasingly use biological and imaging biomarkers (eg, cerebrospinal fluid and positron emission tomography) to determine the patient’s particular risk for developing Alzheimer disease (AD) and other dementias, as well as to differentiate the dementia type [6-8]. Although these biomarkers are an advance to the current diagnostic schemas widely promoted [4,5], these now *conventional* biomarkers face several limitations: they are expensive, difficult to access, invasive or inconvenient, and they do not accommodate a high-frequency measurement strategy. In addition, clinical and neuropsychological assessments, although remaining the core gold standard, are time-consuming, require self-report, and are subject to interassessor variability. More importantly, they are performed at discrete points in time in contexts that can affect their sensitivity (eg, patient comorbid conditions, medications, motivation, etc).

To improve this current clinical paradigm, digital biomarkers provide an alternative and rapidly developing approach. Digital biomarkers are defined here as objective, quantifiable, physiological, and behavioral data that are collected and measured by means of digital devices, such as embedded environmental sensors, portables, wearables, implantables, or digestibles. Digital biomarkers allow objective, ecologically valid, long-term follow-up with frequent or continuous assessment that can be minimally obtrusive or function in the background of everyday activity. Further, these frequent measures can capture intraindividual variability in performance that may be the earliest indicator of change [9-12] and thus detect subtle health transitions (eg, healthy to MCI). Even more potentially transformative, this approach may also allow us to discover novel and innovative digital indicators, such as gait-speed variability over time [11,13] or computer use metadata [10,14].

The adoption of these methodologies has been hampered by a number of factors [15,16]. The approach requires an interdisciplinary team, there is a multitude of sensors and devices that can be used, and there are no agreed-upon standards for these digital biomarkers. Most importantly, there is not a large evidence base indicating which standards are most effective. Much of the literature focuses on a narrow perspective using a single device or technology (eg, a wearable or a cognitive testing app). Most research has been limited to small numbers of participants assessed in a smart apartment or bioengineering laboratory. However, there is a growing movement in this research area to bring the technologies out of the laboratory and to the larger community in so-called “living lab” or “life laboratory” settings. The focus in these settings is to develop and confirm the utility of these technologies in the everyday environment of older adults’ homes. In this review, we take stock of this research to answer the following questions: (1) What is the evidence for real-life, home-based use of technologies for early detection and follow-up of MCI or dementia? And based on this current evidence, (2) What transformation might clinicians expect in their everyday practices?

## Methods

### Information Sources and Study Selection

**Figure 1:**
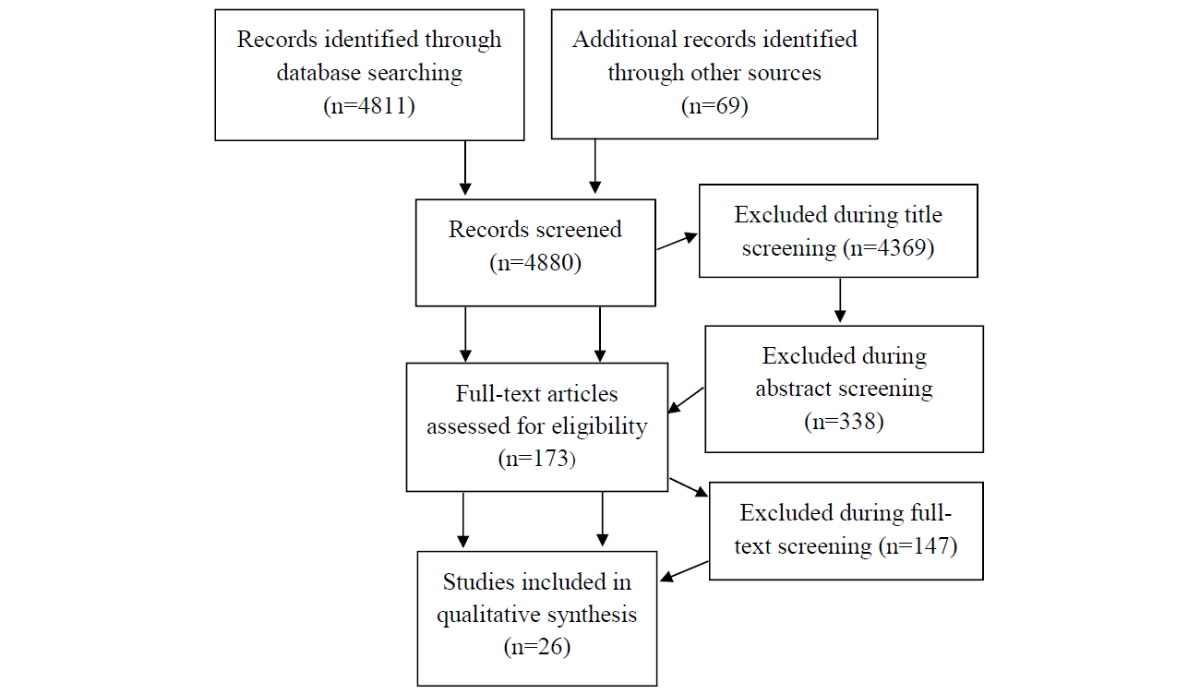
Flow diagram of the study selection process.

We followed the Preferred Reporting Items for Systematic Reviews and Meta-Analyses (PRISMA) statement [17]. A systematic search was conducted of PubMed, including the Institute of Electrical and Electronics Engineers (IEEE); Cochrane; and Scopus databases. We searched for papers published from inception to July 14, 2018, for original research studies examining the implementation of ICT for MCI to mild AD follow-up and detection in real-life settings. We used the following Medical Subject Headings (MeSH) search terms and keywords: “clinical trial,” “evaluation,” “assessment,” “Alzheimer*,” “cognitive impairment,” “MCI,” “dementia,” “cognition,” “technology,” “telehealth,” “telemonitoring,” “e-health,” “internet,” “sensors,” “global positioning system,” “phone,” “smartphone*,” “computer,” “tablet,” and “smart home*.” We updated search terms after an initial review of our search yield. We only considered English-language publications. Additional articles were obtained by scanning reference lists of literature collected on that basis. Two reviewers (AP and KW) conducted initial eligibility screening based on title and abstract, followed by assessment of full-text versions (see Figure 1 for more details). Any disagreements were resolved by consensus after a third opinion (JK).

### Eligibility and Exclusion Criteria

Published studies that included the following were considered for inclusion: community-dwelling older adults (aged 65 years or more); healthy participants, if cognitive status was monitored, or those presenting cognitive decline (ie, from subjective cognitive complaints to early AD); a focus on home-based ICT evaluation for follow-up; and remote diagnosis of cognitive deterioration. Studies were excluded if they did or did not do the following: did not include data-generated results; included only moderate-to-severe AD; focused on caregiver support (eg, social support); focused on behavioral and psychological symptoms of dementia management (eg, global positioning system [GPS]-based wandering tracking); or took place in a controlled area (eg, smart, simulated, apartment laboratory or single-test home). Computerized cognitive tests, which mostly involve online evaluation at discrete points in time without longitudinal continuous assessment, have already been reviewed elsewhere [18,19].

### Search Results

The initial search yielded a total of 4811 records. Articles were screened based on titles and abstracts, of which 173 full-text versions were assessed for inclusion. A total of 26 studies were finally eligible for inclusion in the review. There was an initial disagreement on eligibility between the two reviewers (AP and KW) concerning only 2 studies, which were finally excluded after consensus between three reviewers (JK, AP, and KW) because they evaluated sporadic, computerized, online testing rather than longitudinal follow-up. The most common reasons for exclusion were as follows: inappropriate setting (eg, hospital setting), intervention (eg, drugs and rehabilitation), or population (eg, psychiatry and Parkinson disease). Because of the great heterogeneity across selected studies in this developing research field, we did not perform a meta-analysis.

## Results

An initial sample of 4811 English-language papers were retrieved from three electronic databases. After screening and review, 26 studies were eligible for inclusion in the review (see Tables 1-5) [10-14,20-40]. These 26 studies were observational studies taking place at home with community-dwelling older people, which is in line with the scope of the review. Sample size ranged from 12 to 279 participants. Mean age ranged from 64 to 89 years and percentage of female participants ranged from 49% to 92%. A total of 10 studies were considered comparative studies. Cognitive status was measured, with various methodological quality. There was a wide range of study duration, from 3 days to 3.6 years of follow-up.

We summarized and classified these 26 studies into four groups, although there was overlap, based on the proposed technological solutions to extract relevant data: (1) data from dedicated embedded or passive sensors, (2) data from dedicated wearable sensors, (3) data from dedicated or purposive technological solutions (eg, games or surveys), and (4) data derived from use of nondedicated technological solutions (eg, computer mouse movements). A fifth group includes solutions that fall into more than one category.

**Table 1:** Summary of 9 studies that included data from dedicated embedded or passive sensors in homes and cars (Group 1).

First author (year), country	Technology description	Study description: design; number and type of subjects (number living alone^a^, if relevant) and setting; duration	Cognitive status and number of participants; age; number of male and/or female participants	Main results
Hayes (2008), United States [13]	Infrared motion sensors and magnetic contact door sensors	Comparative observational study; 14 elderly living alone in the community; 6-month follow-up (mean 315 days, SD 82)	Healthy group (n=7, CDR^b^=0, MMSE^c^ ≥24), MCI^d^ group (n=7, CDR=0.5, MMSE ≥24); mean age 89.3 years; 5 males, 9 females	Walking speed and activity of MCI group was more variable than that of the cognitively healthy controls.
Suzuki (2010), Japan [20]	Passive infrared sensors to record in-house movements	Observational study; 50 elderly living alone in the community; 1-year follow-up	MMSE ≥24; mean age 80.9 years; participant gender NC^e^	Association between lower numbers of outings with decrease of indoor movements and cognition declines.
Kaye (2012), United States [21]	Unobtrusively measures every instance of walking past a line of four passive infrared motion sensors fixed sequentially on the ceiling	Observational study; 76 persons living alone and independently; 4-week period	Mean MMSE=28.3; mean age 85.9 years; 86% women	Faster speeds were correlated with better cognitive test scores.
Dodge (2012), United States [11]	Passive infrared sensors fixed in series on the ceiling of the homes	Observational longitudinal study; 93 elderly living alone at home independently; mean follow-up of 2.6 years (SD 1.0)	54 cognitively intact, 8 with aMCI^f^, 31 with naMCI^g^; mean age 84.9, 84.5, and 83.8 years, respectively; 88%, 84%, and 91% women, respectively	Daily walking speeds and their variability are associated with naMCI; naMCI presented a slowing of walking speed over 3 years. The highest and lowest variability were also found to be predominantly associated with naMCI.
Hayes (2014), United States [22]	Infrared motion sensors and magnetic contact door sensors	Comparative, observational, cross-sectional study; 45 elderly living independently and alone; 26 weeks	16 MCI, 29 cognitively intact; mean age 87 years; 89% female	aMCI volunteers had less disturbed sleep than both naMCI and cognitively intact volunteers, as measured by movement in bed, wake after sleep onset, and times up at night.
Petersen (2015), United States [23]	Total out-of-home daily time in hours assessed unobtrusively using an in-home activity sensor platform (eg, infrared sensors in each room and contact sensors on the doors to the home)	Observational study; 85 independent older adults who lived alone; 1 year	75 (CDR=0), 10 (CDR=0.5); mean age 86.4 years; 87% female	More hours spent outside the home was associated with better cognitive function.
Dawadi (2016), United States [24]	Smart homes: combination motion and light sensors on the ceilings and combination door and temperature sensors on cabinets and doors	Observational study; 18 community-dwelling seniors living alone; 2 years	7 cognitively healthy, 6 lowered performance, or cognitive difficulties (1 dementia, 4 MCI) (MMSE NC); age 84.7 years; 5 females, 13 males	Statistically significant correlation between sensor-based daily activity behaviors and clinician-provided cognitive assessment scores.
Urwyler (2017), Switzerland [25]	In-home, wireless, unobtrusive sensors network to detect activities of daily living	Comparative observational study; 20 participants living alone; 20 consecutive days	10 dementia, 10 healthy controls (MMSE=29.1 vs 23.0); age 76.7 vs 73.9 years; 70% female in both groups	Activity differed significantly between the healthy and diseased participants.
Seelye (2017), >United States [26]	Continuous routine driving-monitoring using an unobtrusive driving sensor: passive sensing device plugged into participants’ vehicles data port	Observational study; 28 older adults living at home: 19 of 28 (68%) lived alone; average of 206 days	21 intact cognition, 7 MCI (average MMSE=28.6); mean age 82.0 years; 62% female	MCI participants drove fewer miles and spent less time on the highway per day than cognitively intact participants. MCI drivers showed less day-to-day fluctuations in their driving habits.

^a^The number of participants living alone is specified when the information is relevant; for example, for ambient sensors but not for wearables devices.

^b^CDR: Clinical Dementia Rating.

^c^MMSE: Mini Mental State Examination.

^d^MCI: mild cognitive impairment.

^e^NC: not communicated.

^f^aMCI: amnestic MCI.

^g^naMCI: nonamnestic MCI.

**Table 2:** Summary of 6 studies that included data from dedicated wearable sensors: accelerometers and GPS^a^-based solutions (Group 2).

First author (year), country	Technology description	Study description: design; number and type of subjects (number living alone^b^, if relevant) and setting; duration	Cognitive status and number of participants; age; number of male and/or female participants	Main results
Westerberg (2010), United States [27]	Sleep monitoring with a wrist-worn activity sensor device	Comparative observational study; 20 volunteers; 2 weeks	10 aMCI^c^ patients (MMSE^d^=27.8), 10 controls (MMSE=29.3); mean age 71.1 and 72.5 years, respectively; 8 and 7 females, respectively	Actigraphy parameters failed to reveal significant differences between groups.
Shoval (2011), Israel [28]	Tracking using a location kit: a GPS with radio frequency identification	Observational study; 41 community-dwelling participants; 28 days	13 healthy, 21 MCI^e^, 7 mild dementia (MMSE and CDR^f^ NC^g^); mean age 72.9, 78.3, and 81.9 years, respectively; 54% female	The spatial range of the mobility of elderly people with cognitive impairment is severely restricted, with most out-of-home time spent in close proximity.
Tung (2014), Canada [29]	GPS-enabled mobile phone	Observational comparative study; 52 older adults; 3 days	19 mild-to-moderate AD^h^ (MMSE=23.1), 33 controls (MMSE NC); mean age 70.7 and 73.7 years, respectively; 40% and 64% female, respectively	GPS-derived area, perimeter, and mean distance from home were significantly smaller in the AD group compared to controls.
Wettstein (2015), Germany and Israel [30]	Mobility data: questionnaires and GPS receiver with a global system for mobile communications modem and a monitoring unit in the home	Observational comparative study; 257 older adults; 4 weeks	35 mild AD (mean MMSE=24.1), 76 MCI (mean MMSE=27.0), 146 healthy persons (mean MMSE=28.6); age 74.1, 72.9, and 72.5 years, respectively; 49% female	Questionnaire-based cognitively demanding activities showed a significant difference between MCI and cognitively healthy participants, and a significant difference between AD and cognitively healthy participants.
Takemoto (2015), United States [31]	GPS and accelerometer	Observational study; 279 older adults; 6 days	MMSE NC; mean age 83 years; 71% female	Number, distance, and minutes of pedestrian trips, as well as vehicle trips were not associated with cognitive functioning.
Mancini (2016), United States [32]	Quality and quantity of turning during normal daily activities by wearing three inertial sensors (one on their belt and two on shoes) during the day	Observational study; 35 elderly adults: 16 nonfallers, 12 one-time fallers, and 7 recurrent fallers; 7 days	Nonfallers (MMSE=28.3), one-time fallers (MMSE=28.9), recurrent fallers (MMSE=28.0); age 83.9, 86.0, and 88.4 years, respectively; 66% female	Visuospatial and memory function scores were associated with quality of turning.

^a^GPS: global positioning system.

^b^The number of participants living alone is specified when the information is relevant; for example, for ambient sensors but not for wearables devices.

^c^aMCI: amnestic MCI.

^d^MMSE: Mini Mental State Examination.

^e^MCI: mild cognitive impairment.

^f^CDR: Clinical Dementia Rating.

^g^NC: not communicated.

^h^AD: Alzheimer disease.

**Table 3:** Summary of 6 studies that included data from dedicated or purposive ICT^a^-monitoring solutions, such as phone-based automated interviews, Nintendo Wii, and virtual reality (Group 3).

First author (year), country	Technology description	Study description: design; number and type of subjects (number living alone^b^, if relevant) and setting; duration	Cognitive status and number of participants; age; number of male and/or female participants	Main results
Mundt (2007), United States [33]	Use of IVR^c^ technology (ie, pressing keys) to administer simple cognitive evaluations by phone during a 20-minute, computer-automated telephone call	Observational comparative study; 107 community-dwelling participants; 24 weeks: IVR administered at home at weeks 4, 12, and 20	36 cognitively normal, (MMSE^d^=28.1), 37 MCI^e^ (MMSE=25.6), 34 mild dementia (MMSE=20.0); mean age 76.7 years; 42% female	The automated administration of IVR simple cognitive tests via phone calls reliably and validly discriminated cognitive functioning among normal, MCI, and mild dementia.
Allard (2014), France [34]	Monitoring of behavior, semantic memory performance, and daily life experiences using a personal digital assistant five times a day	Observational study; 60 older adults; 7 days	60 healthy participants (mean MMSE=27.0); mean age 75.1 years; 45% female	Magnetic resonance imagery markers were significantly associated with mobile assessments of semantic memory performance.
Brown (2016), United Kingdom [35]	Touch screen system to assess multiple domains of health and behavior; cognitive tasks scheduled once per day	Observational study; 40 community-dwelling adults; three periods of approximately 7 days	40 healthy participants (mean MMSE=28.63); mean age 72 years; 24 females, 16 males	Convergent validity with, and similar levels of, reliability to the standard cognitive battery.
Seelye (2016), United States [36]	Completion of a short 12-item weekly online questionnaire of health and life events, administered on desktop computers	Observational study; 83 independent, community-dwelling older adults; 1 year	59 healthy (MMSE=28.8), 24 MCI (MMSE=27.4); mean age 86.2 and 87.9 years, respectively; 88% and 75% female, respectively	Online questionnaire performance significantly correlated to cognitive test. MCI participants submitted their questionnaires progressively later in the day and they needed greater assistance from staff as compared with intact participants.
Zygouris (2017), Greece [37]	Tablet personal computer with software enabling the self-administration of a cognitive assessment through virtual reality	Comparative, two-arm, observational study; 12 elderly living at home; 1-month follow-up	6 healthy and 6 MCI; mean 64 years; 3 males, 9 females	Performances to complete the given exercise differed significantly between healthy and MCI groups, yielding a correct classification rate of 92% for MCI detection.
Leach (2018), United States [38]	A Nintendo Wii balance board used to quantify postural sway twice daily, under a single-task condition and under a dual-task condition, using a daily word-search task administered via a Nook tablet	Observational study; 20 healthy community-dwelling elderly; 30 days	Mean MMSE=28.6; mean age 87.0 years; 65% females	Linear relationships were observed between the day-to-day variability in postural sway and cognitive status.

^a^ICT: information and communication technologies.

^b^The number of participants living alone is specified when the information is relevant; for example, for ambient sensors but not for wearables devices.

^c^IVR: interactive voice response.

^d^MMSE: Mini Mental State Examination.

^e^MCI: mild cognitive impairment.

**Table 4:** Summary of 4 studies that included data derived from nondedicated ICT^a^ solutions use, for example, secondary analysis of everyday computer use and pill box use (Group 4).

First author (year), country	Technology description	Study description: design; number and type of subjects (number living alone^b^, if relevant) and setting; duration	Cognitive status and number of participants; age; number of male and female participants	Main results
Hayes (2009), United States [39]	Adherence to a twice-daily vitamin C regimen measured using an electronic 7-day pill box	Observational cross-sectional study; 38 participants living independently in the community; 5 weeks	A high cognitive function group (MMSE^c^=28.8) and a low cognitive function group (MMSE=28.0); mean age 82.8 years; 68% female	The low cognitive function group was significantly less adherent than the healthy elders. Very mild cognitive impairment had a detrimental and significant impact on medication adherence.
Kaye (2014), United States [10]	Remotely monitored computer use	Comparative observational study; 113 elderly living independently and alone or who were the only computer user; mean 36-month follow-up	38 MCI^d^ and 75 cognitively intact; mean age 85 years; 92% female	Decrease in number of days with use, mean daily usage, and an increase in day-to-day use variability in MCI subjects.
Seelye (2015), United States [40]	Mouse pointer movement variables were computed during routine home computer use using algorithms that identified and characterized mouse movements within each computer use session	Observational comparative study; 62 older adults living at home alone or who were the only computer user in the household; 1 week	42 healthy (MMSE=28.8), 20 MCI (MMSE=27.3); mean age 87.9 and 87.5 years, respectively; 88% and 80% female, respectively	MCI was associated with making significantly fewer mouse moves and making mouse movements that were more variable, less efficient, and with longer pauses. Mouse movement significantly associated with several cognitive domains.
Austin (2017), United States [12]	Computer monitoring software used to track the terms people entered while conducting Internet searches as a measure of language and cognition	Observational study; 42 community-dwelling older adults living alone; 6 months	Cognitively intact, with the exception of 1 participant (CDR^e^ score ≥0.5, suggesting MCI); average age 81.1 years; 83% female	Individuals with higher cognitive function used more unique terms per search and employed less-common terms in their searches.

^a^ICT: information and communication technologies.

^b^The number of participants living alone is specified when the information is relevant; for example, for ambient sensors but not for wearables devices.

^c^MMSE: Mini Mental State Examination.

^d^MCI: mild cognitive impairment.

^e^CDR: Clinical Dementia Rating.

**Table 5:** Summary of a solution that falls into more than one category (Group 5).

First author (year), country	Technology description	Study description: design; number and type of subjects (number living alone^a^, if relevant) and setting; duration	Cognitive status and number of participants; age; number of male and/or female participants	Main results
Seelye (2018), United States [14]	Weekly online survey metadata metrics based on survey engagement patterns	Observational study; 110 healthy older adults; 3.6-year follow-up	110 with intact cognition at the beginning and 29 transitioned to MCI^b^ during study follow-up (MMSE^c^=28.8); mean age 84.8 years; 77% female	At baseline, incident MCI participants completed surveys later in the day than cognitively intact participants. Longitudinally, incident MCI participants showed an increase in survey completion time compared with cognitively intact participants.

^a^The number of participants living alone is specified when the information is relevant; for example, for ambient sensors but not for wearables devices.

^b^MCI: mild cognitive impairment.

^c^MMSE: Mini Mental State Examination.

In the first group (ie, embedded dedicated sensors), we can principally cite *smart home* technologies [11,13,20-25] and *smart car* technologies [26]. Studies in the second group (ie, data from wearable dedicated technologies) mainly rely on accelerometers and GPS solutions [27-32]. The third group (ie, dedicated ICT solutions) imply ICT-supported monitoring solutions [33-38]. These mainly employ online surveys or touch-screen tests [34-36] as well as computer-automated telephone calls [33] or a Nintendo Wii-dedicated game [38]. The fourth group (ie, monitoring of nondedicated ICT solutions use) consists of secondary analyses of commonly used technologies, including everyday computer use [10,12,40] and pill box use [39]. The fifth group included one study that dealt with monitoring of dedicated ICT solutions using survey metadata metrics analysis [14].

## Discussion

### Evidence for Real-Life, Home-Based Use of Technologies

The first aim of this paper was to provide an overview of technologies for real-life early detection and follow-up of cognitive function to practicing clinicians involved in management of AD and related disorders. A total of 26 studies were identified, with a variety of technologies and a wide range of study duration and sample size. The first key observation is that compared to the overall number of publications in the field, few papers dealt with home-based, real-world evaluations. Most excluded articles focused on technology functionality, tests of technical aspects in laboratory settings, and focused evaluations in single or a few *test-bed* homes or hospital settings. Most technologies were far removed from everyday life experiences or widely disseminated implementation in the community. Among the included study types, the first (ie, embedded dedicated sensors), the third (ie, dedicated ICT solutions), and the fourth groups (ie, monitoring of nondedicated ICT solutions use) have the common advantage of unobtrusiveness. They rely on everyday life observation without any, or very minimal, participant involvement. The fact that these are among the longest studies in this review, up to several years, likely speaks to the passive nature of the technologies. Several studies in the third group are partly similar to studies of computerized online tests, with the difference that a longitudinal follow-up and a self-administration of nonconventional cognitive tests at home is evaluated [34-36]. In contrast, wearable technology (eg, GPS and wrist-worn device) studies are generally short-term studies. This may be explained by the difficulties in implementing such solutions in real-world settings, as they demand more extensive end-user participation (eg, remembering to wear or charge the device) in this older adult population with various levels of cognitive impairment and technical capacity.

The exclusive use of ambient passive sensors in homes and cars does not guarantee good acceptability to end users. As an example, people may have an intrusive perception of a 3D camera or microphones. However, authors do not report any acceptability issues for experiments involving infrared, temperature, humidity, luminescence, and magnetic door contact sensors or driving sensors [11,13,20-26]. Studies on monitoring the use of personal computers have yielded comparable results [10,12,40]. However, this has only been validated in a select population thus far, as discussed in the Limitations section below, and acceptability outcomes are not always reported.

The completion rate of repeated remote assessments using ICT monitoring solutions was high, generally above 80% [33-35]. Over a longer period of time, Seelye et al [14,36] reported that online weekly health forms were submitted on schedule 75% of the time. Using a Nintendo Wii balance board, Leach and al [38] found an average of 3 days of missing posture and cognitive data over the 30-day observation period for each subject.

For the wearable devices, most studies do not report acceptability issues, such as refusal rate at inclusion and adherence data during follow-up. Mancini et al [32] reported that all 35 participants complied with the protocol (ie, wearing inertial sensors) for 7 consecutive days, while Shoval et al [28] found that participants actively wore the device for 88% of the days. Finally, Wettstein et al [30] reported that the major reasons given to refuse participation in their study were distrust and fear of being observed. In the case of wearables, samples are smaller and/or durations are shorter, which limits comparisons.

### What Transformation Might Clinicians Expect?

With advances in monitoring technologies, we can anticipate what clinicians might expect in their practice in the coming years. A major limitation of all these studies is the selection of volunteers that are relatively homogeneous (ie, white, educated, receptive to technology, and living in urban areas). If, in the future, there is wider use of ICT and digital biomarkers in clinical practice, a perceived advantage of technologies over *traditional* biomarkers, we need to develop ICT in a way that ensures its acceptance and usability under nonoptimal conditions for long periods of time. Most of the solutions presented in these studies are not mature enough for this goal.

The evidence base from dedicated embedded sensors (Group 1) seems to be the most mature research area in this field. There is evidence that these sensor-based technologies are sensitive to detecting cognitive and functional change. Nevertheless, this is still an area of active research, not yet translated into wide clinical adoption. Several advances are needed. Most studies dealing with embedded sensors are limited to participants living alone. It remains technically complex to disambiguate activity in multi-person homes in a real environment. New sensing approaches and data fusion algorithms are in development, but this remains work in progress. Dedicated home systems require installation, which can be a barrier to wider dissemination. Several approaches to this need have been developed, including prepared field kits (ie, “sensors in a box”) for ready deployment [41] and online video installation guides [15]. With these and other advances, one can reasonably anticipate that some of these solutions could be proposed in everyday care to a large population in the coming decade. The Collaborative Aging Research Using Technology (CART) Initiative [42] in the United States is a multi-site, nationwide project that uses multiple embedded sensing technology and diverse data to facilitate research in the field of independence and health of older adults from diverse communities. Funded by the National Institutes of Health and the Department of Veterans Affairs, the CART Initiative has been designed to enable expansion of the home-based sensing platform to 10,000 homes across the United States in several years.

Dedicated wearable sensors (Group 2) have several barriers to overcome before readiness for large community-wide implementation. They have been perceived as obtrusive and stigmatizing. Although their adherence rate may be lower, their great advantages are that their use is not limited to people living alone and they can provide both indoor and outdoor information. We believe, however, that significant progress can be made by improving both their accuracy [43] and their development and evaluation processes. Technical-centered development of these devices in laboratory settings disregards the barriers to successful implementation in real-life situations and, more particularly, acceptability for unselected (eg, low computer literacy and cognitively impaired) populations. This real-life implementation depends on technical improvements for deployment in suboptimal contexts (eg, uncertain Internet coverage) and on the end user’s acceptability. In recent years, several research teams have tried to design creative multiphase studies, applying iterative modifications of a proposed digital technology-associated solution or using participatory design approaches [44,45]. Living laboratory, iterative design approaches, here defined as a “research method which brings together end users, developers, and health professionals in a cocreation and evaluation process,” introduce an intermediate phase closer to *real life* before deployment. Participatory design methodology also emphasizes the involvement of users throughout the innovation process. Widespread diffusion of these concepts in medical research has not yet been achieved. However, health-related functions of dedicated wearable devices have become commonly offered in routine use devices (eg, GPS and accelerometers) and hopefully will be integrated in a more efficient way for health use in the near future [46,47].

Dedicated ICT solutions (Group 3), such as embedded assessment algorithms within home-based cognitive computer games, remains a promising area [48]. Surprisingly, few trials evaluated such solutions in a home environment. Trials involving mobile phone apps are also promising, notably in the psychiatry field [49,50], but they are not at an advanced stage in the dementia research area of interest, other than assessments taking place in a dedicated place with the presence of an operator outside of the home [51-53]. Finally, a promising field is in the monitoring of nondedicated ICT solutions (Group 4), as they have the advantage of unobtrusiveness in common with the embedded dedicated sensors (Group 1), and they do not require the installation of dedicated devices. This area is limited by the willingness of this population to adopt ICT solutions in their everyday lives. Nevertheless, generations follow one another, and future older adults may be more likely to be interested in new technologies. In that case, assessing those solutions in an ideal target population may be seen as less of a limitation if a large dissemination is expected within the next 5-10 years.

The wider adoption for all these technologies will require particular attention to specific use cases, ranging from detecting early cognitive decline to assessing change in function during treatment; ease of deployment; data provenance and analysis; and creating not just evidence for efficacy but evidence for effectiveness. The first drivers of this transformation will likely be the research community and the growing adoption of community-based studies. Clinical trials in particular are an area where this approach is very promising. Often, proof of efficacy in trials leads to practice adoption.

### Limitations

As already stated by Pillai et al [54], keywords describing ICT and AI vary and they are not always specified in the papers, as this research field is not yet mature. Nevertheless, it is unlikely that we have missed many relevant studies, as we chose the broadest possible key search terms and followed up with an extensive hand search of full-text references and key terms. Promising results from studies related to screening and assessment rather than follow-up [55,56], or conducted in controlled settings, are not presented in this paper. All the studies reviewed here were conducted in real-life homes of older adults. Several issues were not highlighted in this paper, including health inequalities [57], ethical issues, data security, information overload for clinicians, and business models of technology implementations, among others. These key issues also need to be addressed early during the evaluation and development process of health technology research, before larger dissemination can occur [58].

### Conclusions and Future Research Directions

The studies included in this review cover a diversity of designs and approaches representing many new avenues of research. There is no conclusive evidence at this stage on the superiority of one or many digital biomarker assessments over others. This is a new area of research. Even for studies based on the same cohorts and, therefore, on comparable populations and locations, the technologies used (eg, driving or computer tracking), sample sizes engaged, and statistical methods differ. For similar outcomes of interest (eg, activity), there is a wide range of digital biomarkers: walking speed, overall activity, outdoor time, etc. It is difficult to know which biomarkers will be most relevant for broader future applications. Further, it is not yet clear which outcomes are best correlated with cognitive decline or, more generally, with mental health. Choices will probably initially focus more on the ease of implementation of a technical solution in a given environment than on direct comparisons as to their accuracy, a comparison that is difficult to make in practice.

Nevertheless, monitoring cognitive and functional domains using ICT devices will grow rapidly and will likely involve AI [59,60] and innovative biomarkers derived from such methods as automated speech and language analysis, motor performance assessments, computer use abilities, and online questionnaire responses and their metadata. These advances will facilitate the transition to proactive, personalized, and participatory medicine. In achieving this goal, the gap between real-life clinical practice and clinical research will be narrowed with clinical trials reflecting patients’ typical activities and outcomes [3,41]. Integration of heterogeneous data (eg, environmental data and multiple biomarkers) will improve our understanding and management of cognitive decline; accordingly, some of these solutions may become adopted into everyday care among the wider population in the coming decade.

## Abbreviations

AD: Alzheimer disease
AI: artificial intelligence
aMCI: amnestic MCI
CART: Collaborative Aging Research Using Technology
CDR: Clinical Dementia Rating
GPS: global positioning system
ICT: information and communication technologies
IEEE: Institute of Electrical and Electronics Engineers
IVR: interactive voice response
MCI: mild cognitive impairment
MeSH: Medical Subject Headings
MMSE: Mini Mental State Examination
naMCI: nonamnestic MCI
NC: not communicated
ORCATECH: Oregon Center for Aging and Technology
PRISMA: Preferred Reporting Items for Systematic Reviews and Meta-Analyses
RFID: radio frequency identification
